# The Prevalence of Alert Pathogens and Microbial Resistance Mechanisms: A Three-Year Retrospective Study in a General Hospital in Poland

**DOI:** 10.3390/pathogens12121401

**Published:** 2023-11-28

**Authors:** Anna Tenderenda, Monika Eliza Łysakowska, Anna Gawron-Skarbek

**Affiliations:** 1 Department of Geriatrics, Medical University of Lodz, 90-647 Lodz, Poland; anna.tenderenda@stud.umed.lodz.pl; 2 Department of Microbiology and Medical Laboratory Immunology, Medical University of Lodz, 90-213 Lodz, Poland

**Keywords:** antimicrobial resistance, alert pathogens, resistance mechanisms, ESBL, *Escherichia coli*, β-lactamase AmpC, MRSA, CPE, VRE, COVID

## Abstract

The development of antibiotic resistance mechanisms hinders the treatment process. So far, there is limited data on the problem of bacterial resistance in hospitals in Central and Eastern Europe. Therefore, this study aimed to assess the prevalence of resistance mechanisms and alert pathogens based on reports regarding cultures of samples collected from general hospital patients in Poland in the period 2019–2021. This study examined the prevalence of resistance mechanisms and alert pathogens and the structure of microorganisms, including the type of diagnostic material in the hospital department. The frequency of occurrence and the trends were analysed based on Cochran’s Q-test, relative change and the average annual rate of change (AARC). Of all 14,471 cultures, 3875 were positive for bacteria, and 737 were characterised by resistance mechanisms (19.0%). Alert pathogens were identified in 983 cases (24.6%), including pathogens isolated from blood samples. The most commonlyisolated bacteria were *Escherichia coli* (>20% of positive cultures), *Enterococcus faecalis* (7%), and *Klebsiella pneumoniae* (6%) increasing over time; *Staphylococcus aureus* (13%) was also found, but its proportion was decreasing over time. Extended-spectrum β-lactamase (ESBL) was the most frequent resistance mechanism in Internal Medicine (IM) (*p* < 0.001) and the Intensive Care Unit (ICU) (*p* < 0.01), as well as in ICU-COVID; this increased over the study period (AARC ↑34.9%). Methicillin-resistant *Staphylococcus aureus* (MRSA) (AARC ↓50.82%) and AmpC beta-lactamase (AARC ↓24.77%) prevalence fell over time. Also, the number of alert pathogens was dominant in the IM (*p* < 0.01) and ICU (*p* < 0.001). The most common alert pathogen was ESBL-positive *E. coli*. Gram-negative rods constitute a significant epidemiological problem in hospitals, especially the growing trend of ESBL in IM and ICU, which calls for increased control of sanitary procedures.

## 1. Introduction

In 1945, during the Nobel Prize lecture, Alexander Fleming warned against the possible consequences of the abuse and improper use of penicillin. These effects were noticeable in the following years, when a rapid increase in staphylococcal resistance was noted [1,2]. Nowadays, in the “post-antibiotic era”, a number of multi-resistant strains have been selected [3,4]. Recent findings indicate that by 2050, deaths due to antibiotic-resistant infections may be even more frequent than deaths due to cancer [5].

Microorganisms can demonstrate either innate (natural) or acquired drug resistance; of these, the latter is more clinically critical [6,7,8] and may be one of the reasons for the lack of effective treatment [9]. This type of resistance develops genetically, either by mutation or the acquisition of new resistance genes through genetic exchange mechanisms [10,11]. Microbial resistance to certain β-lactam antibiotics, such as third-generation cephalosporins and carbapenems, is constantly increasing [12,13,14]. From a clinical point of view, the enzymes capable of hydrolysing most or even all β-lactams, including extended-spectrum β-lactamases (ESBL), carbapenemases-producing *Enterobacteriaceae* (CPE), and metallo-β-lactamases (MBL), have particular importance [15]. It is estimated that about 14% of the global population is colonised by ESBL-producing enteric bacteria, and this value is increasing yearly [16,17]. There is also increasing concern about carbapenems, which have broad-spectrum activity against Gram-negative bacteria such as *Enterobacteriaceae* spp. The first occurrence of the New Delhi metallo-β-lactamase type 1 (NDM-1) strain (*Klebsiella pneumoniae*) in a patient was reported in 2008 [18]. Currently, the genes determining this type of resistance are spreading worldwide [19].

The case of colistin resistance and the related plasmid-mediated mobile colistin resistance MCR-1 gene was first reported in 2016 [20]. Nowadays, the vast spread of colistin resistance among bacteria has great worldwide attention because this last resort antibiotic plays a significant role in treating diseases caused by resistant *Escherichia coli*, *K. pneumoniae*, and *Pseudomonas aeruginosa*, bacteria responsible for common urinary and digestive tract infections [21,22]. Other important resistant pathogens are methicillin-resistant *Staphylococcus aureus* (MRSA) and methicillin-resistant coagulase-negative staphylococci (MRCNS). In addition to the typical MRSA residing in hospitals, new clones can invade community settings and infect people without predisposing risk factors [23,24]. Moreover, macrolide, lincosamide, and streptogramin b (MLSb) resistance is increasingly reported in clinical isolates of Gram-positive bacteria such as *Staphylococcus* spp. and *Streptococcus* spp. [25]. Finally, enterococci may acquire resistance to vancomycin (VRE) and transfer this feature to *S. aureus* [26,27].

Pathogens presenting various resistance mechanisms, and insensitivity to the antibiotics and chemotherapeutics commonly used in therapy, or those responsible for invasive infections, are defined as alert pathogens; these are listed in national and international scientific society guidelines [28,29]. Among others, the following microorganisms are classified as alert pathogens: MRSA, VRSA (vancomycin-resistant *S. aureus*), VRE, ESBL-, KPC-, MBL-positive Gram-negative rods, *Clostridioides difficille,* and all pathogens isolated from bloodstream or cerebrospinal fluid infections [28], (Supplementary data: List of alert pathogens). Other definitions used in the study were provided by the Clinical Laboratory Standards Institute (CLSI), the European Committee on Antimicrobial Susceptibility Testing (EU-CAST), and the United States Food and Drug Administration (FDA) [30,31,32].

There are many potential causes for the growing resistance of bacteria to antibiotics [3,33,34]; as such, there is a need for more reliable data describing the consumption of antibiotics, especially those ordered by primary care physicians. Although many medical professionals underestimate the value of ongoing training in this field, such treatment is one of the most challenging branches of pharmacotherapy, as it is influenced by various factors related to the drug, the patient, and the microorganism [35]. Also, it is important to improve patient knowledge about antibiotics and the natural course of infection: patients often fail to comply with medical recommendations in primary care during antibiotic therapy [36].

For drug resistance to be tackled effectively, a coordinated, multi-sectoral approach is needed [37]. The main areas that require special attention are constant control of nosocomial infections, monitoring of the epidemiological state of the hospital or department, and rational use of antibiotics [36]. However, generally limited data exists regarding bacterial resistance and alert pathogens in hospitals in Central and Eastern Europe.

Therefore, the aim of this retrospective study was to determine the prevalence of microbial resistance mechanisms and alert pathogens in samples collected from patients of a general hospital in Poland. It also examined the prevalence of resistance mechanisms and alert pathogens with regard to patient age, type of diagnostic materials, and hospital department and identified any trends.

## 2. Materials and Methods

### 2.1. Study Design and Data Collection

A retrospective analysis was performed of microbiological test results from diagnostic materials from patients hospitalised in a general hospital (16 departments; 380 beds) (Subcarpathian Voivodeship, Tarnobrzeg, Poland) in 2019–2021. Diagnostic materials were also collected from patients hospitalised in the ICU-COVID department in 2021. In total, 14,471 microbiological tests were analysed. Over 50 different diagnostic materials (typically sent to the microbiology laboratory) were initially subjected to statistical analysis. However, as the data were too dispersed, they were systematised and grouped according to their origin and clinical relevance. The following classification was adopted:

- Skin swabs, including the swabs of wound (superficial, postoperative, post-traumatic swabs), skin lesion swabs, ulceration swabs, pressure ulcer swabs, diabetic foot swabs, burn swabs, navel swabs, and nail plate swabs;
- Body fluid cultures included abdominal fluid, maxillary sinus fluid, pericardial fluid, synovial fluid, pleural fluid, and bile culture;
- Ear cultures, including right and left ear swabs;
- Nasal cultures included nasal swabs on the left and right nostril and nasopharyngeal swabs;
- Throat cultures: tonsil swab, oral swab, and general throat swab;
- Vaginal cultures, including general vaginal swabs and cervical swabs;
- “Other materials”, including the culture of material from the pancreas, a swab from a tumour, a swab from the urethra, and a swab from the fistula.

Other diagnostic materials appearing as independent cultures in the database were left unchanged. Medical documentation and patient data were analysed with complete anonymity. Due to the study’s retrospective nature, informed consent was not required. The study protocol was approved by the Bioethics Committee of the Medical University of Lodz (RNN/184/22/KE).

Bacteria were isolated and identified using standard microbiological and biochemical methods. The Vitek 2 Compact automatic system (BioMérieux, Marcy-l’Étoile, France) was used to identify strains and test susceptibility to various groups of antibiotics. If needed, the data were supplemented with the disc diffusion method (media: BioMérieux, Marcy-l’Étoile, France; antibiotic discs: ArgentaLab, Poznań, Poland). If the tested strain was identified with MRSA or MRCNS phenotype, the disc-diffusion method with the cefoxitin discs (30 μg, Argenta, Poland) was used to confirm such result. MLSb phenotype was confirmed for staphylococci and streptococci with erythromycin (15 μg) and clindamycin (2 μg) discs. If resistance to glycopeptides was detected, the minimal inhibitory concentration of the vancomycin was established with E-tests (Liofilchem, Via Scozia, Italy). ESBL phenotype in Gram-negative rods was confirmed with the disc-diffusion method using discs with amoxicillin/clavulanic acid (20/10 μg), ceftazidime (30 μg), and cefotaxime (30 μg). MBL phenotype was confirmed with the discs with meropenem (10 μg) and meropenem (10 μg)/boronic acid (20 μL). KPC resistance mechanism was confirmed with the discs containing EDTA (10 μL), ceftazidime (30 μg) and imipenem (10 μg). Finally, the OXA-48 phenotype was confirmed with temocillin disc. All the disc diffusion and MIC tests test were carried out on Muller-Hinton Agar II (Argenta, Poznań, Poland) according to EUCAST and KORDLguidelines [38,39,40]. The results were interpreted according to the minimum inhibitory concentration (MIC) interpretive clinical breakpoints recommended by the EUCAST in Breakpoint Tables—v. 9.0 for 2019, v. 10.0 for 2020, v. 11.0 for 2021 [38,39,40]. The quality control strains were *E. coli* ATCC 25922, *P. aeruginosa* ATCC 27853, *K. pneumoniae* ATCC 700603, *Acinetobacter baumannii* ATCC 19606, *S. aureus* ATCC 25923, *S. epidermidis* ATCC 13518, and *Enterococcus faecium* ATCC 29,212 (ArgentaLab, Poznań, Poland). The results, media, and antibiotics were subjected to quality control following the internal laboratory procedures. Each series of antibiotics purchased was tested against recommended reference strains (EUCAST) [38]. Antibiotics often used in the laboratory, e.g., ceftazidime, imipenem, meropenem, vancomycin, or linezolid, were checked every second day against reference strains.

Vitek2 and BacAlert diagnostic devices were validated by the service every 12 months. Confirmatory identification was performed with API strips (bioMerieux, Marcy-l’Étoile, France) in case of doubts about the identification results. E-tests and/or antibiotic discs were used in case of doubts about susceptibility results. Resistance mechanisms were confirmed using E-test strips (resistance to vancomycin, teicoplanin, penicillin) or antibiotic discs (MBL, KPC, OXA-48, MRSA/MRCNS, MLSb mechanisms). To screen the ability of enteric rods to synthesise carbapenemases, the CIM test was performed.

The quality control of the course and results of diagnostic tests using the Vitek 2 device were performed following the manufacturer’s recommendations. The density of each bacterial suspension was tested using a densitometer validated daily and dedicated to the Vitek2 system. Each batch of bacterial identification cards (e.g., GN, GP, YST, and ANC) was tested against the recommended reference strains, as were the cards used for drug susceptibility testing (e.g., P644, P643, N330, YST08).

Alert pathogens were reported based on the Regulation of the (Polish) Minister of Health on “The list of alert pathogens, registers of nosocomial infections and alert factors, and reports on the current epidemiological situation of the hospital” [28].

### 2.2. Microbiological Reports

The database containing the list of performed microbiological tests was prepared using a laboratory IT system (LIS Centrum MARCEL S.A., Zielonka, Poland). The reports included patient age, type of diagnostic material, names of hospital departments, isolated microorganisms, identified resistance mechanisms, and alert pathogens. The prevalence of resistance mechanisms (RM) and alert pathogens (AP) was calculated as a percentage of the total positive cultures (with isolated bacteria or fungi for AP, with bacteria only for RM). Data from the laboratory IT system was transferred to an Excel sheet (Microsoft Office 2019, Microsoft Corporation, Redmond, WA, USA). The collected data was verified by an infection prevention specialist and the head of the medical microbiology laboratory.

### 2.3. Statistical Analysis

Statistical analysis was performed using Statistica version 14 (TIBCO Software Inc., Palo Alto, CA, USA). The *post hoc* multi-comparisons test was used to verify the difference in the number of diagnostic materials collected, resistance mechanisms identified, and the number of alert pathogens between the years of the study. An analysis of variance without repetitions was used to determine the difference in age of patients with identified resistance mechanisms and alert pathogens between the years of the study. The frequency of occurrence of resistance mechanisms year-on-year was analysed with Cochran’s Q test. The level of statistical significance was set at *p* < 0.05.

The following terms were used to describe the direction and dynamics of the observed changes: relative change (RC) and the average annual rate of change (AARC). RC was calculated using the formula:

$$\mathrm{RC}=\;\frac{y_{t}-y_{t-1}}{y_{t-1}}\times 100\%,$$

where y_t_—frequency of occurrence of resistance mechanisms/alert pathogens in the examined year; y_t−1_—frequency of occurrence of the resistance mechanisms/alert pathogens in the preceding year.

AARC was calculated based on the assumption that it increases or decreases consistently each year between the two periods. These calculations were based on the formula:

$$\mathrm{AARC}=(\sqrt[{n}]{\frac{i2020}{i2019}\times \frac{i2021}{i2020}}\times 100\%\;)-100\%,$$

where i—index of prevalence; n—number of terms that are multiplied.

AARC was used to calculate trends in the frequency of isolation of individual microorganisms and trends with the occurrence of resistance mechanisms and alert pathogens in the top five departments which ordered the highest number of cultures.

Since the total number of cases examined in 2020–2021 significantly fell compared to 2019, results were presented using RC and AARC and not only as absolute values; the latter would not be a reliable method because it would not correctly represent the clinical picture. Therefore, it should be remembered that the total number of resistance mechanisms, isolated microorganisms, and alert pathogens may fall each year; however, a decrease in the total number of cultures during the three-year observation may result in a statistically significant increase in the frequency of isolation of the given microorganisms, the occurrence of a resistance mechanism, or alert pathogens.

## 3. Results

### 3.1. General Characteristics

The study covered 14,471 samples of various diagnostic materials from patients hospitalised in 2019–2021 (Table 1). Throughout the study period, the mean age of the patients was 54.6 ± 27.6 years, and the ages ranged from less than one year, including newborns, (*n* = 594; 4.10%) to 104 years. Among the youngest group, 268 (3.99%) were tested in 2019, 166 (4.35%) in 2020, and 160 (4.11%) in 2021. The diagnostic material was collected from 8037 (55.54%) comprised patients aged ≥ 60. The groups included similar numbers of female and male patients (50.14% vs. 49.86%).

Significantly fewer identifications were performed in 2020 and 2021 than in 2019 (Table 1); this number decreased by 43.31% between 2019 and 2020 (*p* < 0.001), but increased slightly (2.01%) between 2020 and 2021 (*p* > 0.05).

#### 3.1.1. Diagnostic Materials

In all analysed years, the top five diagnostic materials were blood samples (3424; 23.7%), urine samples (2804; 19.4%), stool samples (2569; 17.8%), throat swabs (1465; 10.1%), and skin swabs (1048; 7.2%) (*p* < 0.001) (Table 2).

#### 3.1.2. Diagnostic Materials according to Hospital Departments in 2019–2021

During the three-year observation, most of the ordered tests came from the Department of Internal Medicine (IM) (5342; 36.9%), Paediatrics (2738; 18.9%), Intensive Care Unit (ICU) (1030; 7.1%), Surgery (1015; 7%), and Neurology (780; 5.4%). The ICU-COVID ward operated only in 2021; it provided 281 diagnostic materials (1.9%) for the three-year observation period, with 7% of tests ordered in 2021 alone (Table 3). Small numbers of samples were taken in the Rheumatology Department (*n* = 245), the Hospital Emergency Department (*n* = 91), and the Ophthalmology Department (*n* = 10).

The frequency of sample collection in individual wards changed significantly over the three-year observation period (*p* < 0.001).

### 3.2. Isolated Microorganisms

From all the diagnostic materials provided (14,471), bacteria or fungi were found in over 25% of samples (3998; positive cultures) (Table 1). Table 4 lists only the microorganisms with an isolation rate of at least 1% across all three years of the study. Other species are presented in Table S1 (Supplementary).

The numbers of isolated microorganisms changed significantly during the three-years of the study (*p* < 0.001). The most frequently-isolated pathogen was *E. coli*, with a prevalence of over 20% during the three-year observation. The second most frequent was *S. aureus* (approx. 13%), followed by *E. faecalis* (approx. 7%), *K. pneumoniae* (approx. 6%), and *Proteus mirabilis* (approx. 5%). The rarest was *Clostridioides difficile* (approx. 1%).

*E. coli*, *K. pneumoniae*, *P. mirabilis*, *E. faecium*, *Staphylococcus haemolyticus*, and *Serratia marcescens* demonstrated an average annual increase in isolation frequency over the three-year observation period. *Salmonella* spp., *Candida albicans*, and *Haemophilus influenzae* presented a downward trend.

In addition, *S. epidermidis*, *P. aeruginosa*, *Enterobacter cloacae*, *Staphylococcus hominis*, and *C. difficile* did not demonstrate any significant change during the study.

*E. faecalis*, *Streptococcus agalactiae*, *Morganella morganii,* and *A. baumannii* increased in 2021 compared to 2019; *S. aureus* decreased in 2021 compared to 2019 (AARC values marked with * in Table 4).

### 3.3. Alert Pathogens and Mechanisms of Resistance

**Figure 1:**
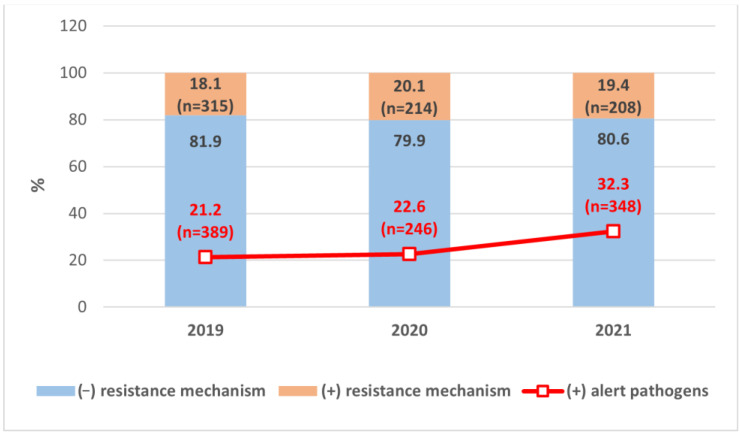
Prevalence of tested resistance mechanisms and alert pathogens among microorganisms isolated in 2019–2021. (−)—lack of resistance mechanism, (+)—presence of resistance mechanism/alert pathogens.

During the three-year study period, approximately 24.6% of isolates were alert pathogens (983 isolates: in 2019, 389; in 2020, 246; in 2021, 348). Their number differed significantly between the analysed years (*p* < 0.001) (Figure 1). The proportion grew over the course of the study: RC increased by 6.59% in 2020 compared to 2019, and by 42.51% in 2021 compared to 2020. In 2021, alert pathogens were isolated 51.91% more often than in 2019. AARC for alert pathogens was 23.25% (*p* < 0.001).

Throughout the study period, resistance mechanisms routinely detected in the microbiological laboratory were confirmed in 19.02% (*n* = 737) of all positive bacterial cultures (*n* = 3875). Fungal isolates (*n* = 123) were not included in the analysis of prevalence of resistance mechanisms. In total, 315 bacterial isolates presenting resistance mechanisms were detected in 2019, 214 in 2020, and 208 in 2021 (Figure 1). The incidence of resistance mechanisms was 11.22% higher in 2020 compared to 2019, and 6.7% lower in 2021 compared to 2020 (*p* < 0.05). The AARC for resistance mechanisms in the study period was 3.53% (*p* < 0.05).

A higher number of alert pathogens was identified compared to resistance mechanisms, mainly due to microorganisms grown from blood cultures, identification of *C. difficile* and other statutorily specified pathogens subjected to reporting as alert pathogens [28].

**Figure 2:**
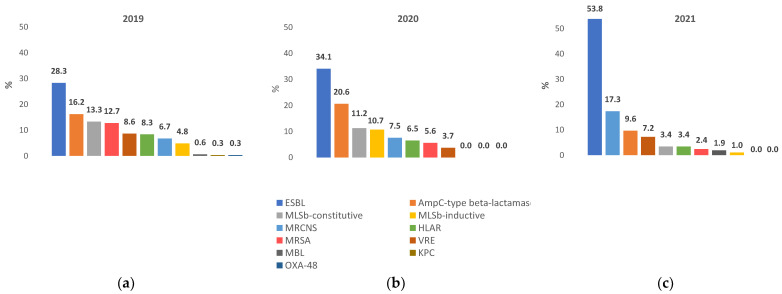
Prevalence of particular resistance mechanisms (**a**) in 2019, (**b**) in 2020, and (**c**) in 2021.

The occurrence of individual resistance mechanisms is presented in Figure 2. Specific upward or downward trends, indicated by the AARC, are given in the text as (↑) or (↓). During the three-year follow-up, the most frequently detected resistance mechanism was ESBL (274 cases in total, in 2019—89 isolates, in 2020—73 isolates, in 2021—112 isolates); ESBL(+) Gram-negative rods increased in prevalence (AARC ↑34.9%). *E. coli* was the most common etiologic factor of urinary tract infections (UTIs), with AARC for ESBL(+) isolates = ↑19.23%. An increase in the proportion of ESBL(+) *K. pneumoniae* was not significant (AARC = ↑0.81%). The three-year mean ESBL prevalence from UTIs was 51.90% for *K. pneumoniae* and 8.77% for *E. coli*. There was a low number of wound and blood isolates in a three-year period (*K. pneumoniae*—14 isolates, and 18 from blood; for *E. coli*—15 and 9 isolates).

AmpC beta-lactamase was more common in 2020 (N = 44) compared to 2019 (N = 51) (RC ↑18.58%); however, RC decreased in 2021 (N = 20) compared to 2020 (↓61.98%), with a three-year AARC of ↓24.77% (115 isolates total). AmpC-positive phenotype was most often detected in the *E. cloacae* complex. The highest proportion of AmpC-positive isolates was detected from wound samples (12 of 22 *E. cloacae* strains in 2019, from other specimens—8 of 22); in 2020, three of nine *E. cloacae* isolates were AmpC positive, in 2021, three of eight. The three-year study prevalence of AmpC-positive *E. cloacae* was 34.43%. In IM and Surgery wards, the prevalence of AmpC-positive strains reached 31.30%, in ICU, 18.26%.

Only two MBL-positive isolates were detected in 2019 (*E. cloacae)* and four in 2021 *(P. aeruginosa*)*;* in 2019 single OXA-48 (*E. cloacae*) and KPC isolates (*E. cloacae*) were observed. Therefore, no trends were observed for CPE in 2019–2021.

The MRCNS mechanism occurred in 74 strains (17.91%) of coagulase-negative staphylococci (in 2019—21 isolates, in 2020—16 isolates, in 2021—36 isolates; AARC ↑29.39%). A fall was observed for gram-positive *Staphylococcus* spp. presenting the MLSb-constitutive mechanism (a total of 47 isolates, AARC ↓51.34%). The MLSb-inductive mechanism was confirmed in a total of 37 *Staphylococcus* spp.; a 132.19% increase was found between 2019 (N = 13) and 2020 (N = 22), while a low number of strains was isolated in 2021 (N = 2, RC ↓88.69; AARC ↓48.77%).

MRSA resistance: in 2019—39 isolates, in 2020—12 isolates, in 2021—5 isolates; a total of 56 strains, AARC ↓50.82%. A total of 37 of 56 MRSA were isolated in three hospital wards (ICU, N = 13; Surgery, N = 14; IM, N = 10). No MRSA were detected in ICU-COVID in 2021. The HLAR resistance mechanism also subsided, being identified in only 50 *Enterococcus* spp. isolates (AARC ↓43.51%).

VRE did not demonstrate any trends with regard to prevalence, but significant variations were observed. VRE was identified in a total of 50 isolates of *Enterococcus* spp. (in 2019—27 isolates, in 2020—8 isolates, in 2021—15 isolates); a 53.45% decrease was found between 2019 and 2020, and there was an increase in 2021 compared to 2020 (↑36.03%; AARC ↓20.39). A total of 31 of 50 VRE was *E. faecium*. Most frequently, VRE was isolated from urine samples (22/50), wound swabs (10/50), and blood samples (12/50). Most VRE were detected in patients over 60 years old.

ESBL—extended-spectrum beta-lactamases; HLAR—high-level aminoglycoside resistance; KPC—*Klebsiella pneumoniae* carbapenemases; MBL—metallo-β-lactamases; MLSb—macrolide, lincosamide and streptogramin b resistance; MRCNS—methicillin-resistant coagulase-negative staphylococci; MRSA—methicillin-resistant *Staphylococcus aureus*; OXA-48—carbapenemase type OXA-48 producing *Enterobacteriaceae*; and VRE—vancomycin-resistant enterococci.

### 3.4. Hospital Department vs. Microorganism

Table 5 presents the structure of the most common microorganisms in the five departments that provided the most diagnostic materials. The most frequent species was *E. coli.* This pathogen occurred at various frequencies in the five selected hospital departments (*p* < 0.001). However, no significant differences in prevalence of *E. coli* were noted with regard to years of the study in the hospital unit.

In the ICU-COVID ward, the most common pathogens were *E. faecalis* (13), *E. coli* (13), *S. epidermidis* (11), and *Staphylococcus haemolyticus* (7) (data are not shown in the table).

### 3.5. Hospital Department vs. Alert Pathogens

During the three-year observation, the most alert pathogens were isolated from diagnostic materials in the IM; in total, 417 alert pathogens were isolated, i.e., 36.04% of all positive cultures in IM (with the exclusion of suspected contaminations). The frequency of alert pathogens increased over the study (*p* < 0.01) (Table 6).

Alert pathogens were also frequently detected in the ICU (*n* = 164), accounting for 43.16% of all positive cultures in ICU. As in the case of IM, the frequency of isolation increased during the three years (*p* < 0.001) (Table 6). In turn, 62 alert pathogens were isolated in ICU-COVID in just one year of the department’s operation.

### 3.6. Hospital Department vs. Resistance Mechanism

Further statistical analysis was conducted for the most commonly identified resistance mechanisms; the number of isolates per year was >0 and simultaneously, they were alert pathogens: ESBL, AmpC, MRSA, and VRE [28]. Analysis included departments that delivered numerous diagnostic materials.

Over the course of the study, the frequency of the ESBL mechanism increased in IM (AARC ↑34.54, *p* < 0.001) and ICU (AARC ↑28.62, *p* < 0.01). The VRE mechanism decreased both in IM (AARC ↓24.28, *p* < 0.001) and in ICU (↓31.08, *p* > 0.05). Although there was a high number of AmpC-positive isolates, no trend was observed over time (*p* > 0.05). The remaining mechanisms appeared with varying frequency in individual departments over time (Table 7).

### 3.7. Age of Patients vs. Prevalence of Alert Pathogens and Resistance Mechanisms

**Figure 3:**
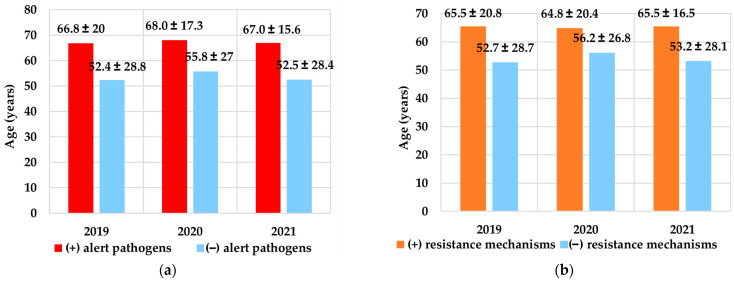
Comparison of the mean ± SD age of the patients in 2019–2021 depending on (**a**) the isolation of the alert pathogens and (**b**) the identification of the resistance mechanism. (+) Alert pathogens—presence of alert pathogens, (–) alert pathogens—lack of alert pathogens, (+) resistance mechanisms—presence of resistance mechanisms, (–) resistance mechanisms—lack of resistance mechanisms, SD—standard deviation.

In all analysed years, alert pathogens were more frequently isolated from older patients (≥60 years) (*p* < 0.001). The mean age of patients from whom alert pathogens were isolated was 67.3 ± 17.6, and those from whom more sensitive microorganisms were obtained was 53.6 ± 28.1 years. A comparison of the mean age of patients with isolated alert pathogens in a given year is presented in Figure 3a.

Similarly, resistance mechanisms were more common in older patients: 65.3 ± 19.2 vs. 53.6 ± 28.1 years (*p* < 0.001) (Figure 3b).

## 4. Discussion

Antibiotic resistance is particularly concerning among microorganisms isolated in a hospital environment. The problem of increasing drug resistance is a public health challenge, and a comprehensive approach and understanding of the issue is needed. Hence, it is necessary to determine the presence of alarm pathogens and mechanisms of resistance in a given medical entity. Also, a rational attitude should be taken towards prescribing antibiotics, considering the analysis of clinical effects and the risk of selection of drug-resistant strains, and regular monitoring must be performed [41].

Multidrug-resistant microorganisms are mainly Gram-negative rods from the *Enterobacteriaceae* family, e.g., *E. coli*, *K. pneumoniae*, *Enterobacter* spp., and *Proteus* spp., and less often, *Serratia* spp. and *Citrobacter* spp. The emergence of strains presenting resistance mechanisms is usually associated with their relatively rapid spread within hospital wards [42,43]. In our study, the most frequently isolated microorganism was *E. coli* (over 20% of all pathogens, N = 905), for which an upward trend was observed in the subsequent years of the research (AARC = ↑10.06). Similarly, *K. pneumoniae* was isolated in around 6% of samples (N = 252), and this value grew over the course of the study (AARC = ↑10.70). The upward trend continued during the COVID-19 pandemic (2020 to 2021), despite a lower number of diagnostic materials collected, suggesting that enteric rods have greater importance in causing infections. Our results correspond to the literature data on increasing levels of Gram-negative bacteria isolation (*E. coli*, *Klebsiella* spp.) in European countries, including bacteria presenting resistance mechanisms [44,45]. Also, Lagacé-Wiens et al. reported significantly higher ESBL production in *K. pneumoniae*, *E. coli,* and moderately, in *E. cloacae* [46]. This trend was also confirmed by other authors and is probably due to the successful spread of ESBL(+) enteric rod clones [47]. The prevalence of *P. mirabilis* was not very high during the study, i.e., from 2019 to 2021 (N = 189, 4.73%); however, the level increased over the three years (AARC = ↑17.09%), which agreed with other results [48].

The most commonly isolated species in most hospital top wards (IM, Surgery, Paediatrics, Neurology) was *E coli*. Also, for all top five wards, the isolation frequency was found to increase, with AARC at over 9% for IM and Paediatrics. On the contrary, Liu and Qin [49] observed fluctuations in the isolation of *E. coli* between 2017 and 2019. Within the top five departments in our study, *S. aureus* was the second most frequently isolated species in all wards, except in the ICU, in 2019 and 2020. Similar results were reported by Liu and Qin et al. [49]. Upward trends were observed for *E. faecalis*, which was in the top four species within the three-year study period in IM, Neurology, and Surgery. In contrast, Liu and Qin [49] observed a decreasing prevalence of enterococci in 2017–2019. The upward trends were also reported for the isolation of *K. pneumoniae* in IM (AARC = ↑20.56%) and ICU wards (AARC = ↑13.12%). Liu and Qin reported *K. pneumoniae* frequently in ICUs, but the number of isolates in their hospital decreased [49].

Among the bacteria isolated from diagnostic materials, the most common were Gram-negative rods. In addition, the isolates presenting ESBL resistance mechanism were most frequently detected (total 274 ESBL-positive; 12.79%). During the three-year follow-up, an upward trend of Gram-negative rods presenting ESBL phenotype isolation was reported (AARC ↑34.9%). A similar increase in the detection of ESBL(+) strains was also observed in Canada, where the prevalence of ESBL(+) *E. coli* increased from 3.9% in 2007 to 12.8% in 2016, and the percentage of ESBL(+) *K. pneumoniae* rose from 1% to 13.6% [46]. Similarly, under the Antimicrobial Testing Leadership and Surveillance (ATLAS) programme, the global prevalence of ESBL(+) *E. coli* was found to be 23.7% and *K. pneumoniae* 35.1% in 2017–2019 [50]. In retrospective studies conducted on children in Shenzhen, China (2014–2018), as much as 49.54% of *E. coli* strains synthesised ESBLs; however, a downward trend was observed over time [45]. In the present study, 82 of 905 *E. coli* isolates (9.06%) were ESBL(+), but this resistance phenotype was more commonly observed in *K. pneumoniae* (113 of 252 isolates; 46.03%). Interestingly, in some healthcare units in Germany, the prevalence of ESBL-positive *E. coli* steadily decreased from 6.4% in 2016 to 4.3% in 2021 [51].

However, most evidence indicates frequent isolation of ESBL(+) strains from hospitalised, immunocompromised patients [52]. In the present study, most ESBL(+) strains were isolated from patients treated in the Internal Medicine ward and ICU; similarly, Meybodi et al. found that this resistance phenotype to be most frequently isolated in the IM ward and Surgery [53]. An increasing trend was observed for ESBL(+) isolates from IM (AARC = ↑34.54, *p* < 0.001) and, to a lower extent, from the ICU (↑28.62, *p* < 0.01). The trend continued despite the existing COVID-19 restrictions in 2020–2021 and the reduced number of microbiological tests. Our results correspond to those obtained previously by Lagace-Wiens et al. (*p* < 0.0001) [46] and highlight the increasing clinical significance of the occurrence of the ESBL mechanism. Surprisingly, during one year of activity in the ICU-COVID, only a few *E. coli* ESBL(+) species (N = 2) were isolated, similar to *K. pneumoniae* (N = 4) and *E. cloacae* (N = 5). A small number of ESBL(+) isolates obtained from the ICU-COVID ward is the reason why no trends were observed. However, some research indicates increasing detection of ESBL(+) enteric rods, which may be attributed to the significant use of empirical antibiotics in COVID-19 patients and the functional disturbances of healthcare services [54].

Lagace-Wiens et al. report an increasing proportion of ESBL-producing isolates in all tested diagnostic specimens, *viz.* urine cultures, respiratory, wound materials, and blood samples from 2007 to 2016 [46]. Our findings also indicate an increasing role played by enteric rods producing ESBLs from these materials, especially *E. coli* and *K. pneumoniae*. *E. coli* was the most common etiologic factor of urinary tract infections (UTIs), and the proportion of ESBL(+) isolates increased between 2019 and 2021 (AARC = ↑19.23%). An increase in the proportion of ESBL(+) *K. pneumoniae* from urine samples was noted, but the change was not significant (AARC = ↑0.81%); however, during the whole study period, the proportion of resistant *K. pneumoniae* was much higher than for *E. coli* isolates, i.e., the three-year mean ESBL prevalence was 51.90% for *K. pneumoniae* and 8.77% for *E. coli*. Although our findings are similar to those of others [46,55], there are higher values for *K. pneumoniae* compared to those reported by Fadlallah and Sokhn [48]. The findings strongly underline the importance of using first-line antimicrobials for uncomplicated UTIs to prevent increased resistance to beta-lactam antibiotics.

Unfortunately, substantial statistical analysis was not possible for wound and blood isolates due to their low number in the three-year period (*K. pneumoniae*—14 isolates, and 18 from blood; for *E. coli*—15 and 9 isolates. Also, ESBL(+) species isolated from other materials were not numerous and were difficult to analyse statistically, as noted in previous findings [46]. However, rising trends concerning resistance to beta-lactams have been reported, and there is an urgent need for adherence to treatment guidelines to prevent further spread.

Other Gram-negative species (*Pseudomonas* and *Acinetobacter*) were rarely grown (N = 6). Such a low number of these isolates was a promising result because it was previously reported that ESBL-producing isolates were often resistant to many other antimicrobial agents (fluoroquinolones, aminoglycosides, third-generation cephalosporins) [56,57].

AmpC-beta-lactamases are clinically important cephalosporinases encoded on the chromosomes of many Gram-negative rods (especially *Serratia*, *Pseudomonas*, *Proteus*, *Acinetobacter*, *Citrobacter*, and *Enterobacter* species); all isolates presenting the AmpC phenotype are classified as alert pathogens [28]. In our research, an upward trend in detection frequency was noted for AmpC beta-lactamase in 2020 compared to 2019 (RC ↑18.58%), but in 2021, RC decreased compared to 2020 (↓61.98%); the 3-year AARC was ↓24.77%, indicating a statistically insignificant downward trend. Similar results have been obtained previously (*p* > 0.05) [46].

The most numerous species found to present an AmpC-positive phenotype was the *E. cloacae* complex. The highest proportion of AmpC-positive isolates was detected from wound samples (12 of 22 *E. cloacae* strains) and “other” specimens (8 of 22 diverse materials). However, in the following years, the number of such strains fell; three of nine *E. cloacae* isolates presented AmpC resistance in 2020 but only three of eight in 2021. Throughout the three-year study, the prevalence of AmpC-positive *E. cloacae* was 34.43%, i.e., more than was reported in a study of the rise of AmpC in trauma patients [58]. Unfortunately, no meaningful statistical analysis was possible due to the small number of AmpC-positive isolates dispersed among different specimens. In the present study, the AmpC resistance mechanism was most commonly isolated in IM and Surgery wards, with the prevalence of AmpC-positive strains at 31.30% in both. This result aligned with other data [58].

Research results indicate an increase in the incidence of *Enterobacteriaceae* isolates producing NDM, KPC, and OXA-48 carbapenemases [59,60]. Similar data were obtained in Poland, and in the rest of Europe (ECDC reports) [61], where the frequency of isolation has been growing for several years [62]. Globally, 1.1% of *E. coli* isolates produce carbapenemases; among them, the most common were NDM (43.8%), KPC (8.3%), and OXA-48 (6.5%) [50]. It was found that 13.3% of *K. pneumoniae* isolates were carbapenemase positive (KPC—30.9%, OXA-48—27.8%, NDM—22.2%); *E. cloacae* isolates demonstrated a low frequency of CRE (3.8% of total isolates), with NDM being the most prevalent (35.9% of carbapenemases), followed by KPC (12.7%) and OXA-48 (8.5%) [50]. Such bacteria were often isolated from the respiratory and urinary tracts of hospitalised patients [63,64]. In a study conducted across Switzerland, carbapenemase-producing bacteria were also often isolated from rectal swabs (58.4% strains), wounds (3.9%), and blood cultures (3.6%). Surprisingly, only single *Enterobacteriaceae* isolates displaying these resistance phenotypes were reported in our study—one MBL and KPC-positive *Enterobacter cloacae* complex strain was isolated in the ICU, and one MBL strain and another OXA-48 *E. cloacae* isolate from the IM ward (3/49, 6.12%), in 2019. Due to the small number of isolates, no statistical analysis considering trends in isolation rates was possible. Carbapenem-resistant *P. aeruginosa* isolates have typically been more frequently identified (20.3%) than *Enterobacterales* globally, and these have often presented VIM, NDM, KPC, and OXA phenotypes [50]. In our study, only four *P. aeruginosa* isolates (8/48; 8.33%) were MBL positive; all were isolated from the ICU in 2021.

*P. aeruginosa* have often been isolated from the respiratory and urinary tracts of hospitalised patients [63,64]. In the present study, most carbapenemase-positive strains were also derived from respiratory tract specimens (N = 5), but also from urine (N = 1) and a rectal swab (N = 1). In a study conducted all over Switzerland, carbapenemase-producing bacteria were often isolated from rectal swabs (58.4% strains), wounds (3.9%), and blood cultures (3.6%). Surprisingly, the low frequency of CPE isolation may be due to prophylactic targeted screening (for patient colonisation by CPE), dedicated nursing staff, isolation of the patient, and the sanitary regimen [65].

Among Gram-positive cocci, the most frequently isolated species was *S. aureus* (N = 527, 13.18%), for which the isolation rate slightly decreased over the three-year study period (AARC = ↓5.06%). Similar results were reported by Liu and Qin (2022), where *S. aureus* accounted for 11% of positive isolations [49]. No significant changes were observed for other staphylococci (−5% < AARC < 5%) except for *S. haemolyticus*, for which the AARC reached ↑43.11%; however, the prevalence of this species was low (1.55%), as noted previously [49]. The third most frequently isolated microorganism, *E. faecalis*, became more prevalent over time (AARC = ↑21.32%); similarly, *E. faecium*, which often causes difficult-to-treat infections, demonstrated an AARC of ↑25.72%. Other studies reported a lower prevalence of two main *Enterococcus* spp. (7.1%) [49] and a falling trend of isolation [46].

The other tested significant resistance phenotype, MRSA, was not found to be very common in the analysed hospital. Worldwide, the number of MRSA to all *S. aureus* infections grew until 2008, when it peaked at 44.2%, and has since decreased [66]. Many African countries, except South Africa, have reported an increase in the prevalence of MRSA infections [67]. Interestingly, an increase in the rate of MRSA isolation was also observed during the COVID-19 pandemic (from 8 to 12%) during one-year follow-up; at the same time, a slight increase was also observed in other Gram-positive cocci, VRE [68]. In the present study, MRSA was rarely isolated (56 isolates, 10.63%) and a downward trend was observed over time (AARC = ↓50.82). Such a low prevalence in our hospital is surprising because a body of evidence indicates a rise in MRSA infections during the COVID-19 pandemic, i.e., from 4.6% to 200% [54]. However, the prevalence of MRSA infections may have been reduced by compliance with hand and environmental hygiene and facemask use. Most MRSA cases (37 of 56 isolates) were isolated only in three hospital wards (ICU, N = 13; Surgery, N = 14; IM, N = 10); however, in general, their number decreased, and no MRSA was confirmed in ICU-COVID in 2021. The prevalence of MRSA was also found to fall over a ten-year period in Canadian hospitals [46].

Staphylococci with MLSb and MRCNS resistance mechanisms are not classified as alert pathogens unless they have been isolated from invasive infections [28]. The MLSb phenotype was not common, being detected in only slightly over 8% of isolates. The MRCNS, which can cause life-threatening cardiac infections [69], were detected in a high proportion among Coagulase-negative staphylococci (18.20%), particularly in *S. epidermidis* and *S. haemolyticus*.

Unfortunately, other Gram-positive cocci, VRE, have also presented a threat to hospitalised patients; infections with VRE have been reported in Europe since 1986, and their prevalence globally has been increasing. In the United States, VRE causes 4% of all healthcare-associated infections (HAIs) and is the second most common pathogen after MRSA [70]. In the tested hospital, VRE isolation decreased from 17.53% in 2019 (N = 27; of all enterococci) to 8.25% in 2020 (N = 8; of all enterococci; AARC—19.78%). This result is a cause for optimism; such pathogens are associated with an increased mortality rate [71,72,73], especially if they resist even last-choice antimicrobials [74,75]. Hence, preventing VRE infections is essential to avoid future challenges in their treatment [76]. According to the other authors, VRE infections (mainly caused by *E. faecium* isolates) were commonly reported in high-risk wards (haemato-oncology, geriatric) [77,78]. In the present study, VREs were primarily isolated in the Internal Medicine, ICU, and surgical wards [79]. EARS-Net data show that the proportion of clinical *E. faecium* resistant to vancomycin isolates from patients with invasive infections has remained stable in some countries (fluctuations < 1%, the Netherlands); it has risen to over 25% in Germany [41]. In the present study, 31 of 50 VRE were *E. faecium*. VRE was most frequently isolated from urine samples (22/50), wound swabs (10/50), and blood samples (12/50), as also noted in a previous retrospective study [79]. Interestingly, most enterococci and VRE isolates were detected in patients over 60 years old, confirming that these pathogens often cause infections in immunocompromised, hospitalised individuals [41]. Some enterococci were also present the HLAR (high level of aminoglycoside resistance) resistance mechanism; these are not alert pathogens, but they may pose problems in treating patients suffering from generalised infections.

## 5. Conclusions

The present single-center study presents a wide range of microbiological characteristics from various isolates obtained from a tertiary hospital in Eastern Poland over a three-year period. Little data exist regarding the epidemiological situation in this region, especially during this period, i.e., a year before the COVID-19 pandemic, and then during the pandemic itself. The most common pathogens were *Enterobacterales*, but Gram-positive cocci, *S. aureus* and *E. faecalis,* were also frequently isolated bacteria.

Despite the sanitary regime, the hospital environment is still not free from alert and resistant pathogens; as such, more efficient sanitary inspections and medical procedures are needed to fight the growing trend of ESBL and other resistance mechanisms, especially in IM and ICU.

## Figures and Tables

**Table 1:** Cultures and the age and sex structure of the study group.

Variable		2019	2020	2021	Total	*p*
Age, years		53.3 ± 28.5	56.7 ± 26.6	53.9 ± 27.8	54.6 ± 27.6	<0.001
Sex	Female, N (%)	3402 (50.43)	1824 (47.70)	2030 (52.04)	7256 (50.14)	NS
	Male, N (%)	3344 (49.57)	2000 (52.30)	1871 (47.96)	7215 (49.86)	NS
Total identifications, N (%)		6746 (46.62)	3824 (26.43)	3901 (26.96)	14,471	<0.001
Positive identifications, N (%)		1832 (27.16)	1087 (28.43)	1079 (27.66)	3998	<0.05

Age is presented as mean ± standard deviation; NS—non-significant.

**Table 2:** Distribution of diagnostic materials submitted for analysis in 2019–2021 according to their total number.

Diagnostic Material	2019	2020	2021	Total
	N (%)	N (%)	N (%)	N
Blood	1354 (20.07)	924 (24.16)	1146 (29.38)	3424
Urine	1205 (17.86)	798 (20.87)	801 (20.53)	2804
Stool	1170 (17.34)	636 (16.63)	763 (19.56)	2569
Throat swab	967 (14.33)	310 (8.11)	188 (4.82)	1465
Skin swab	540 (8.00)	281 (7.35)	227 (5.82)	1048
Pus	188 (2.79)	180 (4.71)	243 (6.23)	611
Rectal swab (CPE)	345 (5.11)	159 (4.16)	84 (2.15)	588
Body fluids for culture	161 (2.39)	123 (3.22)	99 (2.54)	383
Nose swab	211 (3.13)	73 (1.91)	55 (1.41)	339
Vaginal swab	94 (1.39)	77 (2.01)	132 (3.38)	303
BAL	111 (1.65)	86 (2.25)	52 (1.33)	249
Sputum	161 (2.39)	60 (1.57)	25 (0.64)	246
Tracheotomy/endotracheal tube swab	85 (1.26)	12 (0.31)	7 (0.18)	104
Vaginal and rectal swabs (GBS)	33 (0.49)	32 (0.84)	26 (0.67)	91
Catheter	27 (0.40)	22 (0.58)	19 (0.49)	68
Ear swab	32 (0.47)	11 (0.29)	11 (0.28)	54
Cerebrospinal fluid	23 (0.34)	15 (0.39)	12 (0.31)	50
Cannula	13 (0.19)	16 (0.42)	4 (0.10)	33
Drain	12 (0.19)	4 (0.10)	2 (0.05)	18
Eye swab	9 (0.13)	1 (0.03)	4 (0.10)	14
Others	5 (0.07)	4 (0.10)	1 (0.03)	10
Total, N (%)	6746 (100)	3824 (100)	3901 (100)	14,471 (100)

BAL—bronchoalveolar lavage, CPE—carbapenemase-producing Enterobacteriaceae, GBS—Group B Streptococcus.

**Table 3:** Distribution of diagnostic materials according to hospital departments in 2019–2021.

Department	2019	2020	2021	Total
	N (%)	N (%)	N (%)	N
IM	2631 (39.00)	1532 (40.06)	1179 (30.22)	5342
Paediatrics	1397 (20.71)	619 (16.19)	722 (18.51)	2738
ICU	441 (6.54)	278 (7.27)	311 (7.97)	1030
Surgery	453 (6.72)	326 (8.53)	236 (6.05)	1015
Neurology	325 (4.82)	241 (6.30)	214 (5.49)	780
Gynecology	260 (3.85)	219 (5.73)	230 (5.90)	709
Orthopedics	345 (5.11)	113 (2.96)	209 (5.36)	667
Oncology	285 (4.22)	144 (3.77)	173 (4.43)	602
Cardiology	208 (3.08)	122 (3.19)	78 (2.00)	408
ICU-COVID	NA	NA	281 (7.20)	281
Neonatology	125 (1.85)	87 (2.28)	66 (1.69)	278
Otolaryngology	175 (2.59)	60 (1.57)	40 (1.03)	275
Rheumatology	66 (0.98)	49 (1.28)	130 (3.33)	245
Emergency	32 (0.47)	33 (0.86)	26 (0.67)	91
Ophthalmology	3 (0.04)	1 (0.03)	6 (0.15)	10
Total, N (%)	6746 (100)	3824 (100)	3901 (100)	14,471 (100)

ICU—Intensive Care Unit, IM—Internal Medicine, NA—non-applicable.

**Table 4:** Distribution of microorganisms with an isolation rate of at least 1% across all three years of the study.

Microorganism	N (%)			Total, N	The Average Annual Rate of Change (%)
	2019	2020	2021		
*Escherichia coli*	384 (20.96)	247 (22.72)	274 (25.39)	905	↑10.06
*Staphylococcusaureus*	245 (13.37)	152 (13.98)	130 (12.05)	527	↓5.06 *
*Enterococcus* *faecalis*	120 (6.55)	72 (6.62)	104 (9.64)	296	↑21.32 *
*Klebsiella* *pneumoniae*	108 (5.90)	66 (6.07)	78 (7.23)	252	↑10.70
*Proteus mirabilis*	78 (4.26)	48 (4.42)	63 (5.84)	189	↑17.09
*Staphylococcus* *epidermidis*	68 (3.71)	57 (5.24)	50 (4.63)	175	(−)
*Pseudomonas* *aeruginosa*	88 (4.80)	38 (3.50)	48 (4.45)	174	(−)
*Enterobacter* *cloacae*	49 (2.67)	34 (3.13)	33 (3.06)	116	↑7.05
*Staphylococcus hominis*	47 (2.57)	41 (3.77)	26 (2.41)	114	(−)
*Salmonella* spp.	50 (2.73)	22 (2.02)	16 (1.48)	88	↓26.37
*Enterococcus faecium*	29 (1.58)	22 (2.02)	27 (2.50)	78	↑25.79
*Candida albicans*	57 (3.11)	15 (1.38)	5 (0.46)	77	↓61.54
*Streptococcus agalactiae*	34 (1.86)	19 (1.75)	23 (2.13)	76	↑7.01
*Staphylococcus haemolyticus*	19 (1.04)	20 (1.84)	23 (2.13)	62	↑43.11
*Haemophilusinfluenzae*	52 (2.84)	5 (0.46)	1 (0.09)	58	↓82.20
*Morganella* *morganii*	17 (0.93)	21 (1.93)	17 (1.58)	55	↑30.34 *
*Acinetobacter* *baumannii*	17 (0.93)	16 (1.47)	14 (1.30)	47	↑18.23 *
*Serratia marcescens*	8 (0.44)	17 (1.56)	20 (1.85)	45	↑105.05
*Clostridioides* *difficile*	18 (0.98)	10 (0.92)	12 (1.11)	40	(−)
Others	339 (18.50)	165 (15.18)	113 (10.47)	617	↓24.77
Total, N (%)	1832 (100)	1087 (100)	1079 (100)	3998 (100)	

↑—increase, ↓—decrease, (−)—no change, *—change in 2021 compared to 2019.

**Table 5:** The number of the four most frequent microorganisms in the top five hospital departments, i.e., delivering the highest number of diagnostic materials.

Year	2019	2020	2021	The Average Annual Rate of Change (%)
Department	Microorganism (N)			
IM	*E. coli* (163)	*E. coli* (85)	*E. coli* (83)	↑9.54
	*S. aureus* (67)	*S. aureus* (30)	*S. aureus* (23)	↓10.04
	*K. pneumoniae* (47)	*K. pneumoniae* (30)	*K. pneumoniae* (29)	↑20.56
	*E. faecalis* (44)	*E. faecalis* (28)	*E. faecalis* (28)	↑22.38
Neurology	*E. coli* (31)	*E. coli* (18)	*E. coli* (23)	(−)
	*S. aureus* (13)	*S. aureus* (10)	*S. aureus* (8)	↓5.39
	*E. faecalis* (10)	*E. faecalis* (5)	*E. faecalis* (8)	↑7.85
	*P. mirabilis* (9)	*K. pneumoniae* (7)	*K. pneumoniae* (6)	NA
Paediatrics	*E. coli* (56)	*E. coli* (36)	*E. coli* (36)	↑15.57
	*S. aureus* (37)	*S. aureus* (23)	*S. aureus* (9)	↓28.92
	*Salmonella* spp. (21)	*Salmonella* spp. (11)	*Salmonella* spp. (10)	(−)
	*S. pneumoniae* (19)	*S. pneumoniae* (5)	*P. aeruginosa* (5)	NA
ICU	*S. aureus* (19)	*S. aureus* (23)	*S. marcescens* (12)	NA
	*K. pneumoniae* (19)	*K. pneumoniae* (12)	*K. pneumoniae* (17)	↑13.12
	*C. albicans* (17)	*E. coli* (12)	*S. epidermidis* (12)	NA
	*S. epidermidis* (14)	*S. marcescens* (11)	*P. aeruginosa* (13)	NA
Surgery	*E. coli* (61)	*E. coli* (58)	*E. coli* (45)	(−)
	*S. aureus* (34)	*S. aureus* (35)	*S. aureus* (24)	(−)
	*E. cloacae* (15)	*P. mirabilis* (20)	*P. mirabilis* (20)	NA
	*E. faecalis* (22)	*E. faecalis* (15)	*E. faecalis* (19)	↑12.67

ICU—Intensive Care Unit, IM—Internal Medicine, NA—not available, (−)—no change if –5% < AARC < 5%, ↑—increase, ↓—decrease.

**Table 6:** Alert pathogens in the top five hospital departments, delivering the highest number of diagnostic materials, in ICU-COVID.

Department	2019	2020	2021	TPC	Prevalence (Overall) (%)	The Average Annual Rate of Change (%)	*p*
	N						
IM	181	122	114	1157	36.04	↑34.38	<0.01
ICU	57	44	63	380	43.16	↑20.88	<0.001
Surgery	48	34	32	839	13.59	↑(-)	NS
Neurology	20	18	22	220	27.27	↑27.50	NS
Paediatrics	7	1	8	160	10.00	↑41.59	NS
ICU-COVID	NA	NA	62	86	72.09	NA	NA

ICU—Intensive Care Unit, IM—Internal Medicine, NS—non-significant, NA—not available, (-)—no change if –5% < AARC < 5%, TPC—Total number of positive cultures in particular departments.

**Table 7:** Prevalence of resistance mechanisms in the top five hospital departments, delivering the highest number of diagnostic materials and in ICU-COVID.

Department	Year			Prevalence (Overall) (%)	The Average Annual Rate of Change (%)	*p*
	2019	2020	2021			
**IM**						
ESBL	46	42	43	18.04	↑34.54	<0.001
AmpC	14	11	3	3.86	↓33.59	NS
VRE	14	5	5	16.90	↓24.28	<0.001
HLAR	13	10	1			
MRSA	10	0	0			
MLSb-inductive	4	2	0			
MRCNS	3	1	2			
OXA	1	0	0			
MLSb-constitutive	2	4	0			
MBL	1	0	0			
**ICU**						
ESBL	16	8	25	25.52	↑28.62	<0.01
MRSA	8	5	1	29.79	↓3.18	NS
AmpC	6	13	2	10.94	↓40.48	NS
VRE	4	2	5	23.40	↓31.08	NS
MRCNS	4	2	4			
HLAR	3	3	1			
MLSb-constitutive	5	3	0			
KPC	1	0	0			
MLSb-inductive	1	3	1			
MBL	1	0	4			
**Surgery**						
ESBL	13	16	15	8.8	↓0.32	NS
AmpC	18	8	10	7.2	↓30.81	NS
MRSA	8	3	3	10.61	↓25.05	NS
VRE	2	0	2			
MLSb-constitutive	14	5	1			
HLAR	7	0	0			
MRCNS	2	5	2			
MBL	0	0	0			
MLSb-inductive	1	7	0			
**Neurology**						
ESBL	4	6	5	10.79	↑28.31	NS
AmpC	1	3	2	4.32	↑62.64	NS
MRSA	2	0	0			
MLSb-constitutive	2	0	0			
HLAR	1	0	1			
MRCNS	1	0	3			
**Paediatrics**						
MRSA	0	0	0			
MLSb-constitutive	1	1	0			
MLSb- inductive	0	0	0			
VRE	1	0	0			
ESBL	0	0	1			
AmpC	0	0	1			
MRCNS	0	0	0			
**ICU-COVID**						
ESBL			13	40.63		
VRE			2	10.53		
HLAR			4			
MBL			0			
MRCNS			17			

AmpC—beta-lactamase type AmpC, ICU—Intensive Care Unit, IM—Internal Medicine, KPC—*Klebsiella pneumoniae* carbapenemase, NS—non-significant, OXA—carbapenemase type OXA-48. The prevalence of a given resistance mechanism was calculated in relation to the number of microorganisms that could present it.

## Data Availability

The data presented in this study are available on request from the corresponding author.

## Supplementary Materials

The following supporting information can be downloaded at: https://www.mdpi.com/article/10.3390/pathogens12121401/s1, Supplementary data: The list of alert pathogens and Table S1: The other microorganisms.
