# Supplementary material for: The Prevalence of Alert Pathogens and Microbial Resistance Mechanisms: A Three-Year Retrospective Study in a General Hospital in Poland
Source: Pathogens. 2023 Nov 28;12(12):1401. doi: 10.3390/pathogens12121401 (PMC10746124; doi:10.3390/pathogens12121401)
Supplement: Supplementary file 1 [file pathogens-12-01401-s001.zip › Supplementary - Table 4a. The other microorganisms..pdf]

Supplementary

Table S4a. Distribution of the other microorganisms with an isolation rate of below 1% (during the study 2019-2021).

Microorganism	N (%)			Total, N
	2019	2020	2021	
Achromobacter xylosoxidans	3	1	1	5
Acinetobacter haemolyticus	0	0	1	1
Acinetobacter junii	0	0	1	1
Acinetobacter lwoffii	3	0	3	6
Acinetobacter ursingii	1	0	0	1
Adenovirus	0	0	1	1
Aeromonas hydrophila	2	1	0	3
Aeromonas salmonicida	0	1	0	1
Aeromonas species	1	0	0	1
Arcanobacterium haemolyticum	1	0	0	1
Bacillus sp.	2	0	0	2
Bacteroides fragilis	2	6	0	8
Bacteroides ovatus	2	0	0	2
Bacteroides thetaiotaomicron	1	1	0	2
Candida ciferrii	0	0	1	1
Candida dubliniensis	2	0	0	2
Candida glabrata	12	4	0	16
Candida krusei	6	0	0	6
Candida kefyr	3	0	0	3
Candida parapsilosis	5	2	0	7
Candida species	4	0	0	4
Candida tropicalis	2	2	0	4
Citrobacter braakii	1	2	0	3
Citrobacter freundii	6	1	7	14
Citrobacter koseri	3	3	4	10
Clostridium clostridioforme	1	0	0	1
Clostridium perfringens	1	0	1	2
Clostridium bifermentans	1	0	0	1
Clostridium group	1	0	1	2
Corynebacterium amycolatum	1	0	0	1
Enterobacter aerogenes	19	5	9	33
Enterobacter asburiae	1	1	0	2
Enterococcus avium	1	0	1	2
Enterococcus durans	2	0	1	3
Enterococcus hirae	1	0	0	1

Enterococcus gallinarum	1	3	2	6
Ewingella americana	0	0	1	1
Fusobacterium nucleatum	2	0	0	2
Fusobacterium necrophorum	2	1	0	3
Gemella morbillorum	1	0	0	1
Granulicatella adiacens	1	0	0	1
Haemophilus parainfluenzae	0	1	0	1
Klebsiella aerogenes	0	1	0	1
Klebsiella oxytoca	18	10	11	39
Kocuria kristinae	10	3	0	13
Kocuria rhizophila	0	1	1	2
Kocuria rosea	4	1	2	7
Kocuria varians	1	0	1	2
Lactococcus garvieae	1	0	0	1
Lactobacillus species	1	0	0	1
Leclercia adecarboxylata	0	0	2	2
Micrococcus species	2	0	0	2
Micrococcus luteus	1	2	2	5
Moraxella catarrhalis	4	2	0	6
Mycobacterium tuberculosis complex	0	1	0	1
Neisseria animalori	0	2	0	2
Pantoea agglomerans	0	1	0	1
Pasteurella canis	2	0	0	2
Pediococcus pentosaceus	1	0	0	1
Prevotella bivia	0	2	0	2
Prevotella disiens	2	0	0	2
Prevotella melaninogenica	2	1	0	3
Prevotella oralis	2	1	0	3
Prevotella oris	1	0	0	1
Prevotella intermedia	1	0	0	1
Propionibacterium acnes	2	0	0	2
Proteus hauseri	3	2	0	5
Proteus penneri	0	5	0	5
Proteus vulgaris	5	4	3	12
Providencia rettgeri	2	1	1	4
Providencia stuartii	4	0	2	6
Pseudomonas alcaligenes	0	1	0	1
Pseudomonas fluorescens	4	3	0	7
Pseudomonas luteola	0	1	0	1
Pseudomonas putida	1	2	0	3
Pseudomonas stutzeri	1	0	0	1

Raoultella ornithinolytica	0	2	7	9
Raoultella planticola	2	2	1	5
Rotawirus-antygen	4	0	9	13
Saccharomyces cerevisiae	1	1	1	3
Serratia fonticola	0	1	0	1
Serratia liquefaciens	2	1	0	3
Serratia odorifera	2	0	0	2
Serratia plymuthica	1	0	0	1
Sphingomonas paucimobilis	1	0	0	1
Staphylococcus auricularis	3	2	1	6
Staphylococcus capitis	12	12	1	25
Staphylococcus caprae	1	0	0	1
Staphylococcus cohnii	1	0	0	1
Staphylococcus lugdunensis	1	4	1	6
Staphylococcus pseudointermedius	2	0	0	2
Staphylococcus saprophyticus	3	1	2	6
Staphylococcus simulans	1	0	0	1
Staphylococcus warneri	3	7	3	13
Stenotrophomonas maltophilia	5	4	0	9
Streptococcus anginosus	5	0	3	8
Streptococcus constellatus	1	0	0	1
Streptococcus gallolyticus	2	1	1	4
Streptococcus dysgalactiae	2	1	1	4
Streptococcus gordonii	1	0	0	1
Streptococcus group A	22	6	0	28
Streptococcus group B	16	9	2	27
Streptococcus group C	8	7	2	17
Streptococcus group D-non enterococcus	0	1	0	1
Streptococcus group G	12	4	1	17
Streptococcus group F	1	0	0	1
Streptococcus mitis	7	3	5	15
Streptococcus pneumoniae	21	8	4	33
Streptococcus parasanguinis	2	0	0	2
Streptococcus pseudoporcinus	1	0	0	1
Streptococcus pyogenes	8	5	5	18
Streptococcus salivarius	8	1	1	10
Streptococcus sanguinis	2	0	2	4
Veillonella species	2	0	0	2
	**339**	**165**	**113**	**617**
