# Supplementary material for: The Prevalence of Alert Pathogens and Microbial Resistance Mechanisms: A Three-Year Retrospective Study in a General Hospital in Poland
Source: Pathogens. 2023 Nov 28;12(12):1401. doi: 10.3390/pathogens12121401 (PMC10746124; doi:10.3390/pathogens12121401)
Supplement: Supplementary file 1 [file pathogens-12-01401-s001.zip › Supplementary - The list of alert pathogens.pdf]

The list of alert factors (pathogens)

(according to the Notice of the Minister of Health /Poland/, of January 22, 2021, regarding the publication of the uniform text of the regulation of the Minister of Health on the list of alert factors, registers of nosocomial infections and alert factors, as well as reports on the current epidemiological situation of the hospital):

1) *Staphylococcus aureus* resistant to methicillin (MRSA) or glycopeptides (VISA or VRSA) or oxazolidinones;

2) *Enterococcus* spp. resistant to glycopeptides (VRE) or oxazolidinones;

3) *Enterobacteriaceae* spp. producing extended-spectrum beta-lactamases (e.g. ESBL, AMPc, KPC) or resistant to carbapenems or other two drug groups or polymyxins;

4) *Pseudomonas aeruginosa* resistant to carbapenems or other two drug groups or polymyxins;

5) *Acinetobacter* spp. resistant to carbapenems or other two drug groups or polymyxins;

6) pathogenic strains of *Clostridium difficile* and the toxins A and B they produce;

7) *Clostridium perfringens*;

8) *Streptococcus pneumoniae* resistant to third-generation cephalosporins or penicillin;

9) *Candida* spp. resistant to fluconazole or other drugs from the azoles or candines;

10) *Aspergillus* fungi;

11) Rotavirus;

12) Norovirus;

13) Respiratory syncytial virus;

14) Hepatitis B virus;

15) Hepatitis C virus;

16) Human immunodeficiency virus (HIV);

17) Pathogens isolated from blood or cerebrospinal fluid responsible for general or invasive infections.
